# Pre‐hospital transdermal glyceryl trinitrate for transient ischaemic attack: Data from the RIGHT‐2 trial

**DOI:** 10.1111/ene.16502

**Published:** 2024-10-11

**Authors:** Jason P. Appleton, Mark Dixon, Lisa J. Woodhouse, Craig S. Anderson, Sandeep Ankolekar, Lesley Cala, Timothy J. England, Peter J. Godolphin, Kailash Krishnan, Grant Mair, Keith W. Muir, John Potter, Chris I. Price, Marc Randall, Thompson G. Robinson, Christine Roffe, Peter M. Rothwell, Else Charlotte Sandset, Jeffrey L. Saver, A. Niroshan Siriwardena, Joanna M. Wardlaw, Nikola Sprigg, Philip M. Bath, the RIGHT‐2 Investigators

**Affiliations:** ^1^ Stroke Nottingham University Hospitals NHS Trust Nottingham UK; ^2^ Stroke Trials Unit, School of Medicine University of Nottingham Nottingham UK; ^3^ East Midlands Ambulance Service NHS Trust Nottingham UK; ^4^ George Institute for Global Health, Faculty of Medicine University of New South Wales Sydney New South Wales Australia; ^5^ George Institute Peking University Health Science Center Beijing China; ^6^ Neurology Department, Royal Prince Alfred Hospital Sydney Health Partners Sydney New South Wales Australia; ^7^ Department of Neurology King's College Hospital London London UK; ^8^ Faculty of Health and Medical Sciences University of Western Australia Perth Western Australia Australia; ^9^ MRC Clinical Trials Unit, Institute of Clinical Trials and Methodology University College London London UK; ^10^ Centre for Clinical Brain Sciences, Edinburgh Imaging and UK Dementia Research Institute University of Edinburgh Edinburgh UK; ^11^ Institute of Neurology and Psychology University of Glasgow Glasgow UK; ^12^ Bob Champion Research and Education Building University of East Anglia Norwich UK; ^13^ Population Health Sciences Institute Newcastle University Newcastle UK; ^14^ Neurology Leeds Teaching Hospitals NHS Trust Leeds UK; ^15^ Department of Cardiovascular Sciences and NIHR Leicester Biomedical Research Centre University of Leicester Leicester UK; ^16^ Stroke Research in Stoke, School of Medicine Keele University Stoke‐on‐Trent UK; ^17^ Nuffield Department of Clinical Neurosciences, John Radcliffe Hospital University of Oxford Oxford UK; ^18^ Department of Neurology Oslo University Hospital Oslo Norway; ^19^ Research and Development Norwegian Air Ambulance Foundation Oslo Norway; ^20^ Department of Neurology and Comprehensive Stroke Center David Geffen School of Medicine at UCLA Los Angeles California USA; ^21^ Community and Health Research Unit University of Lincoln Lincoln UK

**Keywords:** blood pressure, clinical trial, stroke, transient ischaemic attack

## Abstract

**Background and purpose:**

Ambulance trials assessing interventions in suspected stroke patients will recruit patients with currently active symptoms that will resolve into transient ischaemic attack (TIA). The safety and efficacy of glyceryl trinitrate (GTN) in the pre‐specified subgroup of patients with TIA in the Rapid Intervention with Glyceryl Trinitrate in Hypertensive Stroke Trial 2 (RIGHT‐2) was assessed.

**Methods:**

RIGHT‐2 was a pre‐hospital‐initiated multicentre randomized sham‐controlled blinded‐endpoint trial that randomized patients with presumed ultra‐acute stroke within 4 h of symptom onset to transdermal GTN or sham. Final diagnosis was determined by site investigators. The primary outcome was a shift in modified Rankin Scale (mRS) scores at 90 days analysed using ordinal logistic regression reported as adjusted common odds ratio with 95% confidence intervals (CIs). Secondary outcomes included death or dependence (mRS >2).

**Results:**

In all, 109 of 1149 (9.5%) patients had a final diagnosis of TIA (GTN 57, sham 52) with mean age 73 (SD 13) years, 19 (17.4%) had pre‐morbid mRS >2, and onset to randomization was 80 min (interquartile range 49, 105). GTN lowered blood pressure by 7.4/5.2 mmHg compared with sham by hospital arrival. At day 90, GTN had no effect on shift in mRS scores (common odds ratio for increased dependence 1.47, 95% CI 0.70–3.11) but was associated with increased death or dependence (mRS >2): GTN 29 (51.8%) versus sham 23 (46.9%), odds ratio 3.86 (95% CI 1.09–13.59).

**Conclusions:**

Pre‐hospital ultra‐acute transdermal GTN did not improve overall functional outcome in patients with investigator‐diagnosed TIA compared with sham treatment.

## INTRODUCTION

Pre‐hospital trials involving presumed stroke patients will recruit a mixed population including those with cerebral ischaemia whose symptoms subsequently resolve within 24 h, diagnosed as transient ischaemic attack (TIA). A recent systematic review found 8% of patients recruited into pre‐hospital stroke trials had a final diagnosis of TIA [1], but few trials have reported their recruited TIA population in detail.

The UK‐based Rapid Intervention with Glyceryl Trinitrate in Hypertensive Stroke Trial 2 (RIGHT‐2) assessed transdermal glyceryl trinitrate (GTN) patch versus sham in 1149 patients with presumed, paramedic‐assessed acute stroke within 4 h of onset [2]. Overall, there was a significant interaction by final diagnosis on the effect of GTN on outcome (*p* = 0.014). Here, a pre‐specified subgroup analysis of the 109 (9.5%) RIGHT‐2 participants with a final diagnosis of TIA is presented.

## METHODS

### Study design

RIGHT‐2 was a UK‐based, prospective, multicentre, paramedic‐delivered, sham‐controlled, participant‐ and outcome‐blinded, randomized trial [2, 3, 4, 5]. Patients were eligible if they presented <4 h of presumed stroke symptom onset to a trial‐trained paramedic; had systolic blood pressure ≥120 mmHg; and a Face–Arm–Speech–Time (FAST) score of ≥2. Exclusion criteria included nursing home resident; Glasgow Coma Scale <8/15; hypoglycaemia (<2.5 mmol/L); or seizure. Full inclusion and exclusion criteria are outlined elsewhere [2]. The trial received ethical approval from the National Research Ethics Committee (IRAS: 167115), was adopted by the National Institute for Health and Care Research Clinical Research Network and was registered (ISRCTN26986053). Participants had routine clinical brain imaging assessed centrally using standardized scores.

### Treatment

Patients were randomized 1:1 to transdermal GTN patch (5 mg, Transiderm‐Nitro® 5, Novartis, Frimley, UK) or sham patch (DuoDERM® hydrocolloid dressing, Convatec, Flintshire, UK). The first treatment was administered by a paramedic in the ambulance and three further daily treatments were given in hospital, placed on the shoulder/back and changed daily. The patch was removed if a non‐stroke diagnosis was made (stroke mimic or TIA) or the patient was discharged prior to the end of the 4‐day treatment period.

### Clinical outcome measures

The primary outcome was death and dependence assessed using the seven‐level modified Rankin Scale (mRS) (0, normal, to 6, died) at 90 days by telephone performed centrally by trained assessors masked to treatment allocation [6]. If the participant was unable, information was collected from a relative/carer or by post.

At day 4 (or hospital discharge, if earlier) trial treatment compliance, neurological status, in‐hospital treatments and investigator‐determined final diagnosis were recorded. At day 90, pre‐specified secondary outcomes were collected: Barthel Index ‐ activities of daily living; telephone Mini‐Mental State Examination, Telephone Interview for Cognition Scale‐modified ‐ cognition; animal naming ‐ verbal fluency; health status utility value calculated from the European Quality of Life, 5 dimensions, 3 levels, European Quality of Life visual analogue scale ‐ quality of life; and Zung Depression Score ‐ mood [3, 7]. Home‐time was the number of days between discharge and day 90. Safety outcomes included all‐cause death.

### Statistical analysis

The statistical analysis plan for the whole trial was applied to this pre‐specified subgroup and performed by intention to treat [4]. The primary outcome was shift analysis of the seven‐level mRS using ordinal logistic regression with adjustment for age, sex, pre‐morbid mRS, baseline FAST score, systolic blood pressure and time from onset to randomization, reported as adjusted common odds ratio. The assumption of proportional odds was tested using the likelihood ratio test. Unadjusted, mean, per‐protocol and imputed sensitivity analyses were performed. For hypothesis generation, heterogeneity of the treatment effect on the primary outcome was assessed in pre‐specified subgroups by adding an interaction term to an adjusted ordinal logistic regression model. Other outcomes were assessed using adjusted binary logistic regression, Cox regression, ordinal logistic regression, multiple linear regression and analysis of covariance. A pre‐specified global outcome (comprising ordered categorical or continuous data for mRS, Barthel Index, Zung Depression Score, Telephone Interview for Cognition Scale modified and health status utility value) was analysed using the Wei–Lachin test [8, 9].

## RESULTS

Of 1149 RIGHT‐2 participants, 109 (9.5%) had a final diagnosis of TIA (GTN 57, sham 52). Amongst all patients with acute cerebral ischaemia (ischaemic stroke or TIA), TIA patients represented 15.9% (57/359) and 15.0% (52/347) of GTN and sham groups respectively. Baseline characteristics were balanced between treatment groups (Table A1): mean age 73 (13) years; white race 101 (92%); FAST score 3, 43 (39%); blood pressure (BP) 161 (24)/92 (16) mmHg; time from symptom onset to randomization 80 min (interquartile range [IQR] 49, 105 min); pre‐event mRS >2, 19 (17%), GTN 0 [0, 2] and sham 1 [0, 2]. There were more female participants randomized to sham (28, 54%) than GTN (19, 33%), and more participants randomized to GTN had atrial fibrillation/flutter recorded in the ambulance (11, 24%) than sham (4, 10%).

Following hospital arrival, those randomized to GTN had a significantly lower Glasgow Coma Scale, non‐significant trends to higher FAST and National Institutes of Health Stroke Scale scores, and numerically more total anterior circulation syndromes than sham participants (Table 1). Overall, baseline imaging features of brain frailty [10] were common—cerebral atrophy 99%; leukoaraiosis 45%; old vascular lesion(s) 72%—and when pooled as a brain frailty score (max 3): 2 (IQR 2,3).

**TABLE 1:** Primary outcome and key secondary outcomes.

	*N*	GTN	Sham	OR/MD (95% CI), adjusted	*p* value
*N*	109	57	52		
Day 90 mRS (0–6), primary outcome^a^	105	3 (IQR 1, 3) (*n* = 56)	2 (IQR 1, 3) (*n* = 49)	1.47 (0.7, 3.11)	0.31
Sensitivity analyses					
Unadjusted	105	3 (IQR 1, 3)	2 (IQR 1, 3)	0.96 (0.48, 1.9)	0.91
Mean mRS	105	2.2 (1.6)	2.2 (1.8)	0.36 (−0.05, 0.78)	0.089
mRS > 2 (%)	105	29 (51.8)	23 (46.9)	**3.86 (1.09, 13.59)**	**0.036**
Per protocol	94	3 (IQR 1, 3)	2 (IQR 1, 3)	1.55 (0.71, 3.41)	0.28
Imputation	109	3 (IQR 1, 3)	2 (IQR 1, 3)	1.63 (0.78, 3.42)	0.19
Admission					
NIHSS (/42)	88	3.4 (3.7)	2.3 (2.8)	0.93 (−0.28, 2.15)	0.13
FAST (/3)	89	1.3 (1)	0.9 (1)	0.37 (−0.03, 0.77)	0.067
OCSP, TACS (%)	94	8 (14.8)	2 (5)	3.13 (0.50, 19.79)	0.22
GCS (/15)^b^	103	14.7 (0.7)	14.9 (0.3)	**−0.31 (−0.51, −0.12)**	**0.002**
Day 90					
Death (%)	107	2 (3.5)	3 (6)	–	–
Disposition (/3)^c^	100	1 (IQR 1, 1)	1 (IQR 1, 1)	0.64 (0.1, 4.11)	0.64
EQ‐5D HUS (/1)^d^, ^e^	99	0.6 (0.3)	0.6 (0.4)	−0.01 (−0.12, 0.11)	0.93
EQ‐VAS^d^, ^e^	96	63.7 (25.1)	57.7 (24.6)	3.56 (−5.02, 12.15)	0.42
Barthel Index (/100)^d^	98	86 (26.8)	79.6 (31.7)	2.04 (−5.74, 9.83)	0.61
TICS‐M^d^, ^e^	59	20.1 (7.2)	21 (9.8)	−0.73 (−3.64, 2.17)	0.62
t‐MMSE^d^, ^e^	59	17.5 (5.6)	16.4 (7)	1.14 (−0.99, 3.26)	0.29
Animal naming^d^, ^e^	59	14.7 (7.3)	15.2 (9.3)	−1.41 (−4.81, 2.00)	0.42
Zung Depression Scale (/100)^d^, ^e^	69	43.4 (20)	52.7 (24.7)	**−8.95 (−17.4, −0.54)**	**0.037**
Home‐time (days)	97	93.2 (32)	96.7 (29.8)	−5.76 (−17.2, 5.64)	0.32
Global analysis, Wei–Lachin^e^	59	–	–	−0.04 (−0.23, 0.15)	0.68

*Note*: Data are number (%), median (interquartile range) or mean (standard deviation). Comparison by binary logistic regression, Cox proportional hazards regression, ordinal logistic regression or multiple linear regression, with adjustment for age, sex, pre‐morbid mRS, FAST, pre‐treatment systolic blood pressure and time to randomization (unless stated). The effect of treatment for GTN versus sham is shown as common odds ratio, odds ratio, hazard ratio or mean difference, with 95% confidence intervals.

Bold indicates *p*<0.05.

Abbreviations: EQ‐5D HUS, European Quality of Life, 5 dimensions, 3 levels health utility status; EQ‐VAS, European Quality of Life visual analogue scale; FAST, Face–Arm–Speech–Time test; GCS, Glasgow Coma Scale; GTN, glyceryl trinitrate; MD, mean difference; mRS, modified Rankin Scale; NIHSS, National Institutes of Health Stroke Scale; OCSP, Oxfordshire Community Stroke Project; OR, odds ratio; TACS, total anterior circulation syndrome; TICS‐M, Telephone Interview Cognition Scale Modified; t‐MMSE, telephone modified Mini‐Mental State Examination.

^a^ Increased odds ratio, i.e. >1, indicates a shift to worse functional outcome.

^b^ Analysed using non‐parametric regression.

^c^ Disposition: home (score of 1), institution or in hospital (score of 2), died (score of 3) by day 90.

^d^ Death assigned: Barthel Index 5, animal naming 1, EQ‐VAS 1, home‐time 1, t‐MMSE 1, TICS‐M 1, EQ‐5D HUS 0, GCS 2, NIHSS 43, Zung Depression Score 102.5.

^e^ Some participants with poor outcomes or dysphasia could not answer cognition, quality of life and mood questions.

Compliance with the first treatment patch was 98%, 29% on day 2, with only 5% receiving all 4 days of trial treatment. Main reasons for non‐compliance were non‐stroke diagnosis (47, 43%) and hospital discharge (22, 20%). GTN lowered BP by 7.4/5.2 mmHg at hospital arrival, but thereafter there was no difference in BP between treatment groups. No participants with TIA received thrombolysis or mechanical thrombectomy.

**FIGURE 1:**
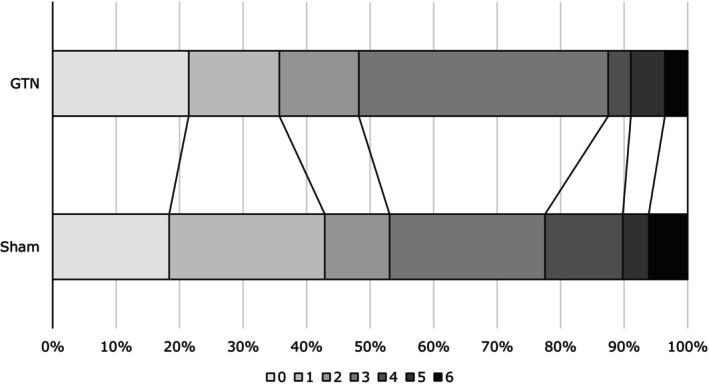
Shift in modified Rankin Scale in 109 participants with a final diagnosis of transient ischaemic attack by treatment group—glyceryl trinitrate (GTN) versus sham. Comparison by ordinal logistic regression with adjustment for age, sex, pre‐morbid modified Rankin Scale, Face–Arm–Speech–Time test, pre‐treatment systolic blood pressure and time to randomization. The effect of treatment for GTN versus sham is shown as adjusted common odds ratio 1.47, 95% confidence interval 0.70–3.11, *p* = 0.31.

The primary outcome (mRS) was available in 105 (96%) participants at day 90. The proportional odds assumption was not violated (*p* = 0.70). There was no difference between GTN and sham groups in the shift analysis: GTN 3 (IQR 1, 3) versus sham 2 (IQR 1, 3), common odds ratio 1.47, 95% confidence interval 0.70, 3.11, *p* = 0.31 (Table 1, Figure 1). There were no statistically significant interactions of the effect of GTN in pre‐specified subgroups. More patients randomized to GTN were dead or dependent (mRS >2) at day 90 than those randomized to sham: GTN 29 (51.8%) versus sham 23 (46.9%), odds ratio 3.86, 95% confidence interval 1.09–13.59, *p* = 0.036 (Table 1). These results were not altered by adding atrial fibrillation into statistical models.

Other outcomes at days 4 and 90 did not differ between GTN and sham, except mood which was better at day 90 in those randomized to GTN (Table 1).

## DISCUSSION

In this pre‐specified subgroup analysis of the RIGHT‐2 trial, 109 participants had an investigator‐determined final diagnosis of TIA. Transdermal GTN lowered BP at hospital arrival, but did not affect the primary outcome of mRS at day 90.

Two phase III pre‐hospital trials have assessed transdermal GTN in presumed ultra‐acute stroke and found no overall benefit [2, 11], with signals suggesting that very early treatment with GTN in severe stroke could be harmful [9, 11, 12]. In contrast, the direction of treatment effect favoured GTN in mimics [13]. Lowering BP acutely during a TIA episode may compromise cerebral blood flow, extending any ischaemic insult, leading to worse clinical outcomes at hospital admission and extended to 90 days. Although current guidelines do not cover acute BP management in TIA, it is possible that subgroups may warrant different BP management strategies similar to ischaemic stroke [14].

Overall, TIA participants had >60 min of symptoms with a demonstrable neurological deficit on hospital admission (mean National Institutes of Health Stroke Scale 3), although none received reperfusion therapies, perhaps due to their mild deficit. Length of hospital stay was >2 days; half were dependent at 90 days, with significant disability, cognitive impairment, reduced quality of life and low mood. Having a presumed transient event was not benign, perhaps reflecting their baseline clinical and brain frailty and potential for deconditioning in the context of an acute illness such as TIA.

There are limitations. First, although this subgroup analysis was pre‐specified, there was no separate statistical analysis plan. Instead, the analyses followed the plan for the overall trial as done previously for the other diagnostic groups [9, 12, 13]. Second, the clinical diagnosis of TIA was determined by site investigators and not centrally adjudicated, so some may have had an alternative diagnosis diluting any effects seen. Third, some TIA diagnoses may have been rendered using the time (symptoms <24 h) rather than tissue (symptoms <24 h and no new infarct) definition and would be considered ischaemic stroke under the tissue approach. Furthermore, the designation of TIA was made post‐randomization and so could represent an outcome; that is, randomized treatment may have shifted participants from being minor strokes to severe TIAs or the reverse. Fourth, the high burden of brain frailty despite being independent according to baseline mRS may have limited any potential treatment effect on outcome. Given the challenges and inaccuracies with using mRS as a pre‐stroke assessment tool, brain frailty could be used as a surrogate for baseline function, predicts clinical outcome after stroke, and could be used in stratification at randomization and/or adjustment in analyses of future stroke trials [10, 15]. Last, the small sample size of this subgroup analysis without adjustment for multiplicity of testing means the findings may reflect chance (particularly since some outcomes went in opposite directions), or measured/unmeasured baseline imbalances.

In summary, ultra‐acute transdermal GTN given in the ambulance to patients with investigator‐diagnosed TIA lowered BP by hospital arrival, but did not influence the shift analysis of mRS at day 90.

## AUTHOR CONTRIBUTIONS

**Jason P. Appleton:** Writing – original draft; investigation; project administration. **Mark Dixon:** Investigation; project administration. **Lisa J. Woodhouse:** Methodology; data curation; writing – review and editing; formal analysis. **Craig S. Anderson:** Writing – review and editing; funding acquisition. **Sandeep Ankolekar:** Writing – review and editing. **Lesley Cala:** Writing – review and editing; investigation. **Timothy J. England:** Funding acquisition; writing – review and editing. **Peter J. Godolphin:** Methodology. **Kailash Krishnan:** Writing – review and editing. **Grant Mair:** Writing – review and editing. **Keith W. Muir:** Writing – review and editing; funding acquisition. **John Potter:** Funding acquisition; writing – review and editing. **Chris I. Price:** Funding acquisition; writing – review and editing. **Marc Randall:** Funding acquisition; writing – review and editing. **Thompson G. Robinson:** Funding acquisition; writing – review and editing. **Christine Roffe:** Funding acquisition; writing – review and editing. **Peter M. Rothwell:** Writing – review and editing. **Else Charlotte Sandset:** Funding acquisition; writing – review and editing. **Jeffrey L. Saver:** Funding acquisition; writing – review and editing. **A. Niroshan Siriwardena:** Funding acquisition; writing – review and editing. **Joanna M. Wardlaw:** Funding acquisition; writing – review and editing. **Nikola Sprigg:** Funding acquisition; writing – review and editing. **Philip M. Bath:** Funding acquisition; writing – review and editing; conceptualization; methodology; investigation; supervision; visualization.

## FUNDING INFORMATION

UK Dementia Research Institute; British Heart Foundation (Grant/Award Number CS/14/4/30972); Nottingham University Hospitals NHS Trust (Grant/Award Number Research and Innovation Award); Stroke Association (Grant/Award Number SA L‐SMP 18/1000).

## CONFLICT OF INTEREST STATEMENT

JPA is supported, in part, by a Nottingham University Hospitals Research and Innovation Award. PMB is Stroke Association Professor of Stroke Medicine and an Emeritus NIHR Senior Investigator. TGR is an NIHR Senior Investigator. GM is the Stroke Association Edith Murphy Foundation Senior Clinical Lecturer (SA L‐SMP 18\1000). JMW is supported by the UK Dementia Research Institute which receives its funding from DRI Ltd, funded by the UK Medical Research Council, Alzheimer's Society and Alzheimer's Research UK. The remaining authors report no conflicts of interest.

## Data Availability

The data that support the findings of this study are available from the corresponding author upon reasonable request.

## Appendix

**TABLE A1:** Baseline ambulance and hospital admission characteristics of TIA patients enrolled in the RIGHT‐2 trial.

	All	GTN	Sham
Ambulance data (pre‐randomization)			
Number of patients	109	57	52
Age (years)	73 (13)	73 (13)	74 (13)
Sex (male) (%)	62 (57)	38 (67)	24 (46)
OTR (min)	80 (IQR 49, 105)	80 (IQR 49, 101)	77 (IQR 49, 112)
<60 min (%)	39 (36)	19 (33)	20 (38)
60–120 min (%)	47 (43)	27 (47)	20 (38)
>120 min (%)	23 (21)	11 (19)	12 (23)
ECG, AF/flutter (%)	15 (18)	11 (24)	4 (10)
Systolic blood pressure (mmHg)	161 (24)	161 (23)	161 (26)
Diastolic blood pressure (mmHg)	92 (16)	91 (16)	92 (17)
Heart rate (bpm)	78 (15)	76 (13)	81 (16)
Glasgow Coma Scale	15 (1)	15 (1)	15 (0)
Glasgow Coma Scale <14 (%)	6 (6)	6 (11)	0 (0)
FAST score (/3)	2 (1)	2 (0)	2 (1)
FAST score = 3 (%)	43 (39)	23 (40)	20 (38)
Hospital admission data (post randomization)			
Ethnic group, non‐white (%)	8 (7)	5 (9)	3 (6)
Pre‐morbid mRS >2 (%)	19 (17)	9 (16)	10 (19)
Medical history (%)			
Hypertension	64 (59)	35 (63)	29 (56)
Diabetes mellitus	20 (19)	10 (18)	10 (19)
Previous stroke	23 (21)	10 (18)	13 (25)
Ischaemic heart disease	19 (18)	12 (21)	7 (13)
Smoking, current	14 (16)	6 (13)	8 (20)
Alcohol, high	4 (5)	2 (5)	2 (6)
Antithrombotic therapy (%)			
Antiplatelets	30 (45)	15 (41)	15 (50)
Anticoagulants	11 (17)	8 (22)	3 (10)

*Note*: Data are number (%), median (IQR) or mean (standard deviation).

Abbreviations: AF, atrial fibrillation; ECG, electrocardiogram; FAST, Face–Arm–Speech–Time test; GTN, glyceryl trinitrate; IQR, interquartile range; mRS, modified Rankin Scale; OTR, onset to randomization; TIA, transient ischaemic attack.
